# Production of the Catechol Type Siderophore Bacillibactin by the Honey Bee Pathogen *Paenibacillus larvae*

**DOI:** 10.1371/journal.pone.0108272

**Published:** 2014-09-19

**Authors:** Gillian Hertlein, Sebastian Müller, Eva Garcia-Gonzalez, Lena Poppinga, Roderich D. Süssmuth, Elke Genersch

**Affiliations:** 1 Institute for Bee Research, Department of Molecular Microbiology and Bee Diseases, Hohen Neuendorf, Germany; 2 Technische Universität Berlin, Institut für Chemie, Berlin, Germany; 3 Freie Universität Berlin, Institute of Microbiology and Epizootics, Berlin, Germany; Goethe University Frankfurt, Germany

## Abstract

The Gram-positive bacterium *Paenibacillus larvae* is the etiological agent of American Foulbrood. This bacterial infection of honey bee brood is a notifiable epizootic posing a serious threat to global honey bee health because not only individual larvae but also entire colonies succumb to the disease. In the recent past considerable progress has been made in elucidating molecular aspects of host pathogen interactions during pathogenesis of *P. larvae* infections. Especially the sequencing and annotation of the complete genome of *P. larvae* was a major step forward and revealed the existence of several giant gene clusters coding for non-ribosomal peptide synthetases which might act as putative virulence factors. We here present the detailed analysis of one of these clusters which we demonstrated to be responsible for the biosynthesis of bacillibactin, a *P. larvae* siderophore. We first established culture conditions allowing the growth of *P. larvae* under iron-limited conditions and triggering siderophore production by *P. larvae*. Using a gene disruption strategy we linked siderophore production to the expression of an uninterrupted bacillibactin gene cluster. *In silico* analysis predicted the structure of a trimeric trithreonyl lactone (DHB-Gly-Thr)_3_ similar to the structure of bacillibactin produced by several *Bacillus* species. Mass spectrometric analysis unambiguously confirmed that the siderophore produced by *P. larvae* is identical to bacillibactin. Exposure bioassays demonstrated that *P. larvae* bacillibactin is not required for full virulence of *P. larvae* in laboratory exposure bioassays. This observation is consistent with results obtained for bacillibactin in other pathogenic bacteria.

## Introduction

Western honey bees (*Apis mellifera*) are important pollinators in many natural and especially agricultural ecosystems [1], [2]. In particular if other pollinating insects like wild bees are missing, managed honey bee colonies are indispensable [3] turning them into the single most important pollinator in agriculture and one of the most important productive livestock [4]. Thus, pathogens and parasites, which cause weakening or death of individual bees and eventually entire colonies, have an impact which reaches far beyond apiculture [5], [6].

The worldwide distributed, notifiable epizootic American Foulbrood (AFB) is one of the few bee diseases which are able to cause the collapse of entire colonies if unnoticed or left untreated. For diagnosed cases of AFB, most authorities consider burning of diseased colonies the only effective control measure which in the end also leads to the loss of the colonies. Since all over the world AFB outbreaks are rather prevalent, this bacterial disease causes considerable economic losses every year. The causative agent of AFB, *Paenibacillus larvae* [7], is a Gram-positive, spore-forming bacterium infecting the honey bee brood only. First instar larvae are most susceptible to the disease and they become infected by ingestion of larval food contaminated with spores of *P. larvae*. These spores are the only infectious form of *P. larvae*. After being swallowed they germinate within approximately 24 hours in the larval midgut [8]. The vegetative bacteria massively proliferate in the midgut lumen until finally they breach through the peritrophic matrix and the midgut epithelium [8]–[10]. The diseased larva dies and larval biomass is converted by *P. larvae* to bacterial biomass.

The species *P. larvae* can be genotyped by repetitive element PCR (rep-PCR) using primers for enterobacterial repetitive intergenic consensus (ERIC) sequences [11] which results in four genotypes, ERIC I – IV [7] which were recently shown to be also discriminable by intact cell mass spectrometry [12]. These genotypes have been demonstrated to differ in several phenotypic features including virulence [13], [14] and expression of virulence factors [15]–[17]. The genotypes *P. larvae* ERIC I and II are frequently isolated from AFB outbreaks all over the world [12], while genotypes ERIC III and IV exist as few historical isolates in different national culture collections (reviewed in [18]). Recently, the genomes of two *P. larvae* strains representing *P. larvae* ERIC I and II have been sequenced, annotated and compared with each other [19]. This analysis confirmed and extended our knowledge on the genetic differences between the genotypes [20] and suggested that these two genotypes developed different strategies to accomplish the same goal, killing the honey bee larvae because *P. larvae* as obligate killer depends on the death of the infected larva for transmission. Although the exact steps of this process are still largely unknown, the past decade has seen considerable progresses in understanding the molecular basis of this disease.

Iron is a micronutrient essential for nearly all organisms, and is involved in numerous processes such as nucleotide synthesis, oxygen metabolism and amino acid production (reviewed in [21]). Although iron is one of the most abundant metals, it is hardly accessible to organisms under aerobic conditions due to its occurrence in the ferric form (Fe^3+^) and the insolubility of its oxides (10^−9^ M Fe^3+^ in soil and water). Hence, iron is a limiting factor for all aerobic organisms; therefore, nature evolved sophisticated means for iron uptake and storage. Honey bees are no exception to the rule as revealed by several studies on iron uptake and storage in bees. In adult honey bees iron rich granules are formed by the accumulation of holoferritin molecules [22], which might either serve as iron storage [22] or might be part of the magnetoreception system [23], [24]. In bee larvae such granules have not been found [25], [26]. Transferrin expression has been studied in both adult and larval honey bees. It was shown that transferrin is expressed at basal levels from 2^nd^ to 4^th^ instar larvae before it increases in 5^th^ instar larvae. Transferrin was shown to be differentially expressed during pupae development [27]. Preliminary studies on the role of iron storage during infection have also been conducted. Artificially challenging adult bees via injection of *E. coli* resulted in only a slight up-regulation of transferrin expression [28] suggesting either that the chosen experimental design was not suitable to analyse all aspects of the bees' response to infection or that infection by Gram-negative bacteria does not influence transferrin expression in adult bees. Transferrin expression in larvae during experimental or natural infection with *P. larvae* has so far not been investigated.

Likewise to the above role for bees, iron is also a limiting factor for growth of aerobic bacteria. In host-pathogen interactions it often plays a crucial role because pathogen and host both compete for the scarce Fe^3+^ ions. To this end, many pathogenic bacteria produce and secrete high-affinity small molecule iron chelators, termed siderophores, which serve as iron scavengers to meet iron limitation by sequestering iron from the host. These low molecular weight iron chelators [29], [30] according to their functional groups are assigned to three types: catecholates, α-hydroxy carbolates, and hydroxamates [31], [32]. The most common siderophore in Gram positive bacteria is the catechol bacillibactin, produced by *Bacillus subtilis*, *B. cereus*, *B. anthracis*, *B. thuringiensis* and *B. amyloliquefaciens*. [33]–[35]. Other catechol type siderophores are paenibactin produced by *Paenibacillus elgii* [36] and the *B. anthracis* petrobactin [37]. While bacillibactin and paenibactin contain 2,3-dihydroxybenzoic acid (DHBA), petrobactin contains the unusual 3,4-dihydroxybenzoic acid as chelating subunit.

Unlike ribosomal synthesis which uses mRNA as the template for the synthesised peptide, non-ribosomal peptide synthetases are megaenzymes commonly organized in modules, each of them delivering one amino acid to the growing peptide chain (reviewed in [38]). Each NRPS-module consists of a basic set of three domains with a dedicated function for the incorporation of an amino acid: the adenylation (A), thiolation (T), and condensation (C) domains. The A-domain specifically recognises and activates an amino acid in an ATP-dependent manner by transformation into the aminoacyl adenylate; the T-domain covalently binds the activated amino acid as a thioester via a phosphopantetheinyl arm and the C-domain catalyses the formation of a peptide bond between two amino acids of two adjacent modules. A terminal module commonly contains an additional thioesterase (TE) domain which releases the peptide from the NRPS. Substrate specification is mainly determined by the A-domain. Known substrates of NRPS comprise not only the set of 20 proteinogenic amino acids but also modified amino acids, nonproteinogenic amino acids, fatty acids, carboxy acids, and several hundred more substrates. Hence, biosynthesis products of NRPS are structurally diverse compounds with many different biological functions like antimicrobial, antiviral, antitumor, immunosuppressive or cytotoxic activities.

Genes encoding for proteins involved in the synthesis of the enzymes of the NRPS complex are often arranged in gene clusters and expressed as operons. Recently, four gene clusters coding for such multienzyme complexes have been identified in the genome of *P. larvae* [19]; one of which was shown to encode for the biosynthesis machinery of sevadicin, a new antibacterially active tripeptide [39]. Another one was shown to encode a complex hybrid NRPS/polyketide synthase (PKS) responsible for the production of paenilamicin [40] which exhibited antibacterial, antifungal, and cytotoxic activity [41] and was demonstrated to be involved in reducing the potentially competing bacterial population present in larval gut [40].

*P. larvae* as a facultative anaerobic bacterium [42] should have strategies to achieve iron supply from the environment including its host during its aerobic life style. Hence, it was not surprising that the most recent *in silico* analysis of two *P. larvae* genomes revealed the presence of a non-ribosomal peptide synthetase gene cluster presumably coding for the biosynthesis of a siderophore. It was proposed that this synthetase will produce a homolog of paenibactin [19], a siderophore produced by *P. elgii* 69 [36]. However, functionality of the cluster as well as identity and function of the product remained elusive.

Herein, we present our results on the identification and characterization of a siderophore produced by *P. larvae*. Using culture conditions optimized for the production of siderophores by *P. larvae*, we demonstrated that *P. larvae* produces a siderophore. Using a gene disruption strategy we linked siderophore production to an NRPS gene cluster recently identified in the *P. larvae* genome by *in silico* analysis [19]. Furthermore, mass spectrometric analysis revealed that the produced siderophore is identical to bacillibactin, a catechol-type siderophore produced by members of the *B. cereus sensu lato* group and *B. subtilis*. Exposure bioassays demonstrated that *P. larvae* bacillibactin is not required for full virulence of *P. larvae* in larval exposure bioassays although a role of iron and of bacillibactin for growth of *P. larvae* under certain conditions could be established.

## Results

### *In silico* analysis of a non-ribosomal peptide synthetase (NRPS) gene cluster putatively involved in the production of a siderophore

**Figure 1:**
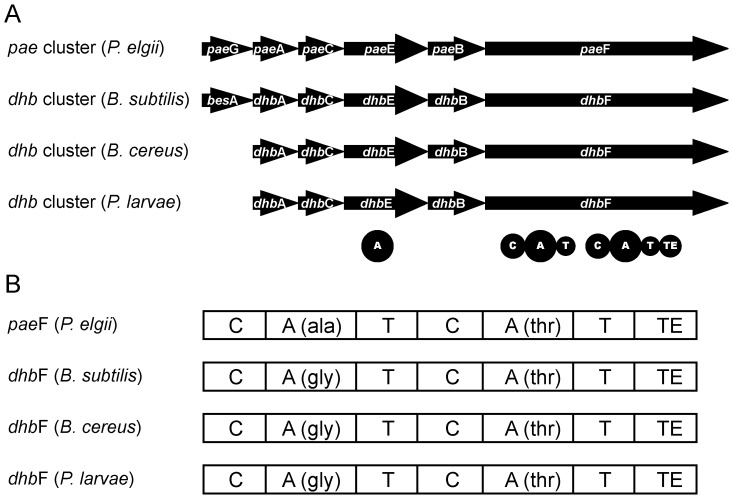
The *dhb* gene cluster of *P. larvae*. (A) Gene arrangement of the *dhb* gene cluster of *P. larvae* in comparison to the *dhb* gene clusters of *B. subtilis* and *B. cereus* involved in the synthesis of bacillibactin and of the *pae* gene cluster of *P. elgii* involved in the synthesis of paenibactin. (B) Domain arrangement within the dimodular genes *pae*F (*P. elgii*) and *dhb*F (*B. subtilis*, *B. cereus*, *P. larvae*) and the predicted amino acids activated by the A-domains. A, adenylation domain; C condensation domain; T, thiolation domain; TE, thioesterase domain. Domain prediction was performed using SBSPKS [44].

Recently, a non-ribosomal peptide synthetase (NRPS) gene cluster putatively resulting in the production of a bacillibactin-like siderophore had been annotated in the genomes of two *P. larvae* strains representing the genotypes ERIC I and ERIC II [19]. This NRPS cluster comprises approximately 11 kB and consists of five genes transcribed in the same direction, *P. larvae dhb*A, *dhb*C, dhb*E*, dhb*B*, and dhb*F* (Fig. 1; GenBank acc. nos. see Table 1), which have been annotated to encode for a 2,3-dihydro-2,3-dihydroxybenzoate dehydrogenase, an isochorismate synthase, a DHBA-AMP-ligase, an isochorismatase, and a dimodular peptide synthetase, respectively (Table 1) [19]. The genomic organization of the *P. larvae* siderophore synthesis cluster showed high similarity to the bacillibactin gene clusters of the *Bacillus cereus sensu lato* group [43] and of *B. subtilis* [35] and the paenibactin biosynthetic cluster of *Paenibacillus elgii* 69 [36] (Fig. 1).

**Table 1:** Similarities between proteins encoded by the *P. larvae dhb* gene cluster (*P. larvae*) and the *B. cereus dhb* gene cluster compared by BLASTX [74].

P. larvae-gene	GenBank DSM25719 (ERIC I)	GenBank DSM25430 (ERIC II)	Amino acid ERIC I	Amino acid ERIC II	Proposed function	Sequence similarity	Similarity (%)
dhbA	ETK27271.1	AHD05396.1	261	261	2,3-Dihydro-2,3-DHBA- dehydrogenase	DhbA, B. cereus	74
dhbC	ETK27270.1	AHD05397.1	408	408	Isochorismate synthase	DhbC, B. cereus	70
dhbE	ETK27269.1	AHD05398.1	541	541	DHBA-AMP ligase	DhbE, B. cereus	79
dhbB	ETK27268.1	AHD05399.1	308	308	Isochorismatase	DhbB, B. cereus	73
dhbF	ETK27267.1	AHD05400.1	2387	2387	NRPS	DhbF, B. cereus	71

Further *in silico* analysis now revealed that the DHBA-AMP-ligase is a unimodular peptide synthetase of 541 amino acids (60 kDa) with one adenylation (A) domain (Table 1). The amino acid substrate specificity of the A domain was predicted to activate 2,3-dihydroxybenzoate (DHB) (Fig. 1) using the web-based software tools “SBSPKS” [44] and “NRPSpredictor2” [45], [46]. The dimodular peptide synthetase encoded by *P. larvae*, *dhb*F (Fig. 1, Table 1), comprises 2387 amino acids in length (267 kDa). Both modules show the typical domain structure with C-, A-, and T-domains. The A-domain of the first module was predicted to activate the amino acid glycine by the algorithms of SBSPKS and NRPSpredictor2. The specificity-conferring amino acid sequence (DILQVGLIWK) was identical to DhbF1 of *B. subtilis* [35]. The second A-domain was predicted to activate the amino acid threonine. The active site residues (DFWNIGMVHK) were identical to DhbF2 of *B. subtilis* [35]. In addition, the second module contained a C-terminal TE domain indicating that this is the terminal module of the NRPS cluster which typically catalyses the final cleavage of the peptide in non-ribosomal peptide synthesis [47], [48]. Hence, our *in silico* analyses suggested that the *P. larvae* siderophore may consist of DHB, glycine, and threonine and form a trilactone (DHB-Gly-Thr)_3_ by intramolecular cyclotrimerization identical to bacillibactin produced by several *Bacillus* species. Our hypothesis that *P. larvae* encodes a peptide synthetase with bacillibactin as biosynthesis product was further substantiated by BLASTX analysis which revealed that on the protein level *P. larvae* DhbACEBF and *B. cereus* DhbACEBF show between 70% and 79% amino acid sequence similarity (Table 1).

### Identification of siderophore production by *P. larvae*

**Figure 2:**
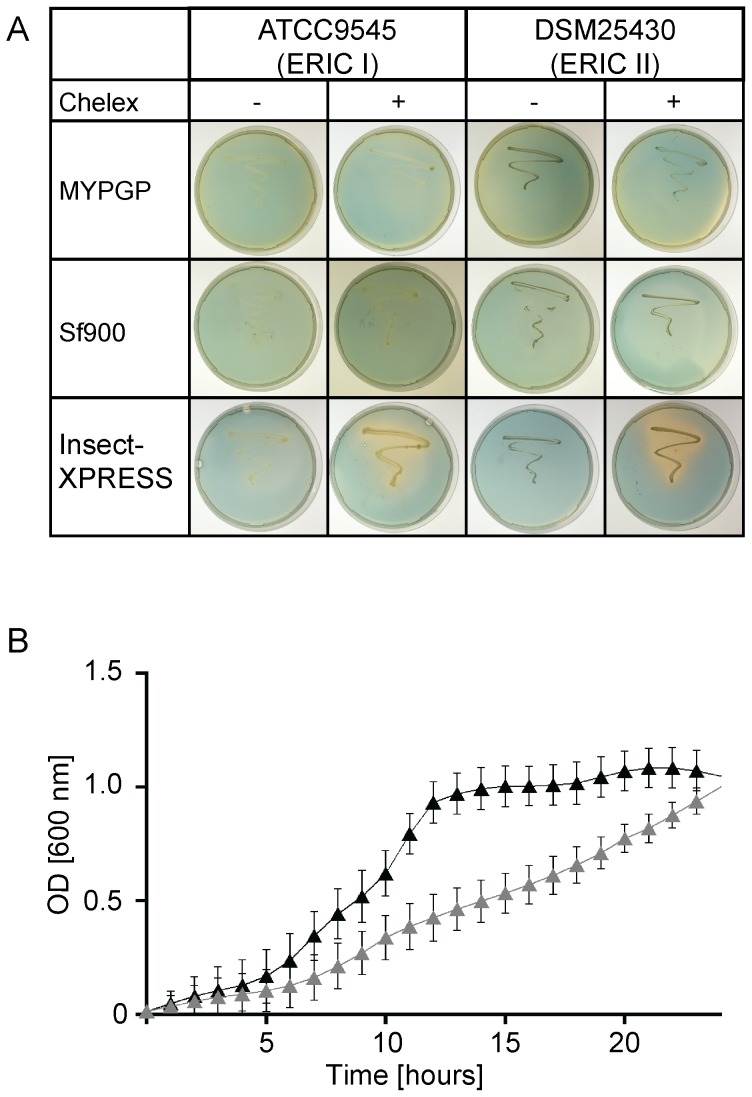
Siderophore production in *P. larvae*. (A) Overlaid CAS agar plate assays for detection of siderophore production by differentially cultured *P. larvae* strains. One colony, each of ATCC9545 (ERIC I) or DSM25430 (ERIC II), was streaked out on different agar plates prepared from MYPGP, Sf900 and Insect-XPRESS media without (-) and with (+) Chelex 100 pre-treatment. Plates were incubated for 72 h at 37°C. Subsequently, the plates were overlaid with CAS agar and incubated for another 2 h. An orange halo around the bacteria grown on Chelex 100 pre-treated Insect-XPRESS agar plates (lower row) indicated the production of a siderophore. (B) Growth kinetics of *P. larvae* in Insect-XPRESS medium without and with pre-treatment with Chelex 100. Bacterial growth was monitored over 24 hours by measuring the optical density at 600 nm (OD_600_) every hour. Three independent experiments (biological replicates) with four technical replicates each were performed; a representative curve is shown. Error bars represent SD from mean values.

In order to confirm functionality of the *P. larvae dhb* gene cluster and analyse siderophore production by *P. larvae*, we established culture conditions triggering siderophore secretion. We first tested common culture media for *P. larvae* (MYPGP, BHI, R2A) and restricted the availability of iron by pre-treating these media with Chelex 100 (BioRad, München, Germany), a chelating ion exchange resin which possesses a high affinity to divalent metal cations, such as Cu^2+^, Zn^2+^, Fe^2+^, Mn^2+^ and also Fe^3+^. All three tested media supported growth of *P. larvae*, irrespectively of whether or not the media had been pre-treated with Chelex 100. However, overlaid CAS agar plate assays [49] did not reveal siderophore production by *P. larvae* under these conditions (Table 2, Fig. 2). Next, we tested different defined media containing only traces of iron (M9; LIM, low iron medium; GMM, glucose minimal medium), which had been used previously in various studies on bacterial siderophore production [50]–[52], in order to trigger siderophore secretion by *P. larvae*. None of these media allowed growth of *P. larvae* (Table 2). Based on an earlier report on the ability of *P. larvae* to grow in EX-CELL405, a serum-free insect cell culture medium [53], we next tested some insect cell culture media already successfully used for culturing honey bee cells [16], [54], [55]. Except for L15 medium, *P. larvae* grew well in these media, even after pre-treatment of the media with Chelex 100 to achieve iron-limited conditions (Table 2). Screening for siderophore production via overlaid CAS agar plate assays revealed that no siderophore production was observed in iron depleted BM3, Schneider's Drosophila and Sf900 II SFM medium. However, *P. larvae* grown in low-iron Insect-XPRESS medium secreted siderophores (Table 2) as indicated by the orange halo around the bacteria streaked out on the otherwise blue colored overlaid CAS agar plates (Fig. 2A). This colour change suggested the production of a siderophore able to remove iron from CAS dye. Analysis of growth kinetics of *P. larvae* in Insect-XPRESS medium revealed that iron-depletion negatively affected *P. larvae* growth (Fig. 2B) suggesting a relevant role of iron for *P. larvae* growth under certain conditions.

**Table 2:** Media tested for the growth of *P. larvae* under low iron conditions and siderophore production by *P. larvae* as revealed by CAS agar plate assays.

Medium	Chelex treatment	Growth of *P. larvae*	siderophore production (CAS assay)	Source
**MYPGP (Mueller-Hinton-yeast-phosphate-glucose-pyruvate)**	No	Yes	-	[7]
	Yes	Yes	-	
**BHI (Brain-heart-infusion)**	No	Yes	-	[70]
	Yes	Yes	-	
**R2A (Reasoner's 2A)**	No	Yes	-	[73]
	Yes	Yes	-	
**M9**	No	No	n. a.	[79]
**LIM (Low iron medium)**	No	No	n. a.	[51]
**GMM (glucose minimal medium)**	No	No	n. a.	[72]
**L15 (Leibowitz-15)**	No	No	n. a.	[54]
**BM 3**	No	Yes	-	[54]
	Yes	Yes	-	
**Schneider's Drosophila Medium**	No	Yes	-	Thermo Fisher
	Yes	Yes	-	(Schwerte, Germany)
**Sf900 II SFM**	No	Yes	-	Thermo Fisher
	Yes	Yes	-	(Schwerte, Germany)
**Insect-XPRESS**	No	Yes	-	Biozym (Hessisch
	Yes	Yes	+	Oldendorf, Germany)

Note: -, not detectable; +, detectable; n.a., not applicable.

### Confirmation of siderophore production by the *dhb* gene cluster

**Figure 3:**
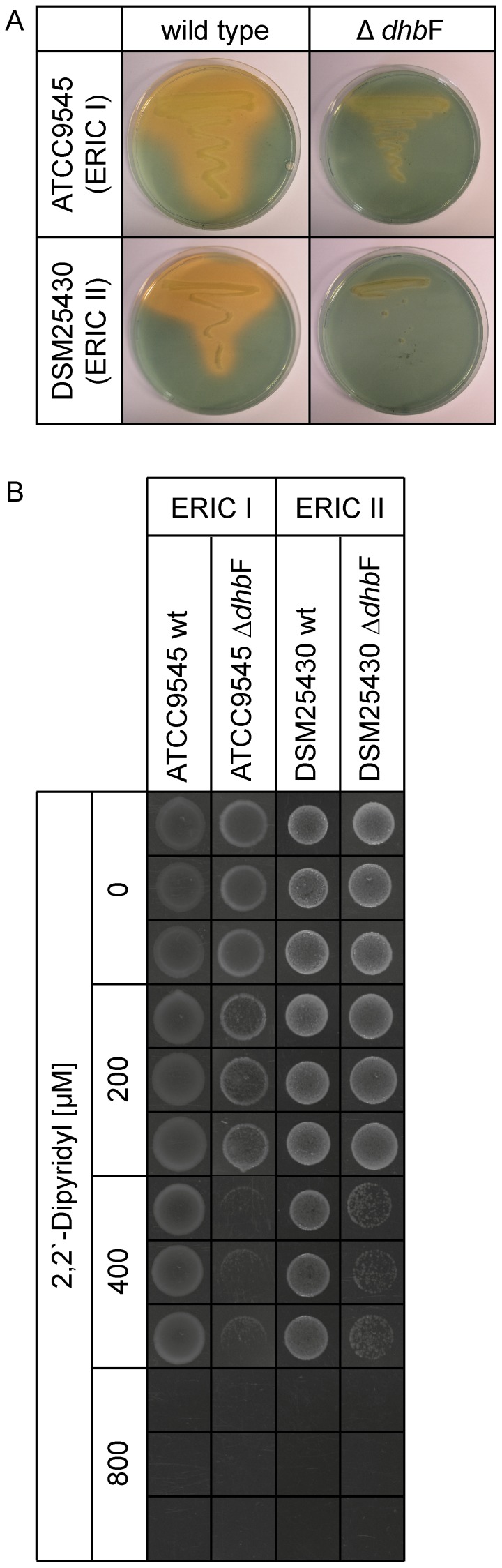
Linking siderophore production and *dhb* gene cluster expression in *P. larvae*. (A) Overlaid CAS agar plate assays for detection of siderophore production in the wild type strains ATCC9545 and DSM25430 and in the corresponding mutant strains ATCC9545 Δ*dhb*F and DSM25430 Δ*dhb*F. Bacteria (one colony of each strain) were streaked out on agar plates prepared from Insect-XPRESS medium pre-treated with Chelex 100. Plates were incubated for 72 h at 37°C. Subsequently, the plates were overlaid with CAS agar and incubated for another 2 h. An orange halo around ATCC9545 and DSM25430 wild type bacteria indicated the production of a siderophore. This halo is missing in the corresponding mutant strains lacking bacillibactin expression thus linking siderophore synthesis to expression of the *P. larvae dhb* cluster. (B) Growth on MYPGP agar plates supplemented with increasing concentrations of the iron chelator 2,2′-dipyridyl (0 – 800 µM) of the wild type strains ATCC9545 and DSM25430 and the corresponding mutant strains ATCC9545 Δ*dhb*F and DSM25430 Δ*dhb*F. 10 µl of the cell suspension were spotted onto the plates, dried for 15 minutes, and incubated at 37°C for 72 h. For each culture, three biological replicates with three technical replicates each were performed. Representative data is shown.

In order to link *P. larvae* siderophore synthesis to expression of the *P. larvae dhb* cluster, gene inactivation mutants deficient in DhbF expression were constructed for each genotype (ATCC9545 Δ*dhb*F, DSM25430 Δ*dhb*F) using a recently published gene disruption strategy for *P. larvae* [15], [16], [39]. For disruption of the *dhb* gene cluster we chose the NRPS gene *dhb*F as the central gene of the operon. Wild type strains *P. larvae* DSM25430 and ATCC9545 as well as the corresponding mutant strains were grown in iron-depleted Insect-XPRESS medium and tested in overlaid CAS agar plate assays. While both wild type strains showed siderophore production, the mutant strains lacked this activity as revealed by the missing orange halo surrounding the bacteria streaked out on the agar plates (Fig. 3A). These results conclusively linked expression of the *dhb* gene cluster to the presence of siderophore production under iron deprivation thus confirming that the *dhb* cluster is responsible for *P. larvae* siderophore assembly.

The mutants were then tested for their ability to grow under iron-restricted conditions, i.e. on MYPGP agar plates supplemented with increasing concentrations of 2,2′-dipyridyl (Fig. 3B). In the absence of 2,2′-dipyridil, growth of both wild-type and mutant bacteria on MYPGP agar plates was unaffected. Growth of ATCC9545 Δ*dhb*F was already slightly affected in the presence of 200 µM 2,2′-dipyridil. Both Δ*dhb*F-mutants showed reduced growth, compared to the corresponding wild-type strains, in the presence of 400 µM 2,2′-dipyridil while growth of both wild-type and mutant strains were equally affected by 800 µM 2,2′-dipyridil. These results reinforced our finding that iron plays a significant role for *P. larvae* growth under certain conditions.

### Identification of *P. larvae* bacillibactin by mass spectrometric analysis

**Figure 4:**
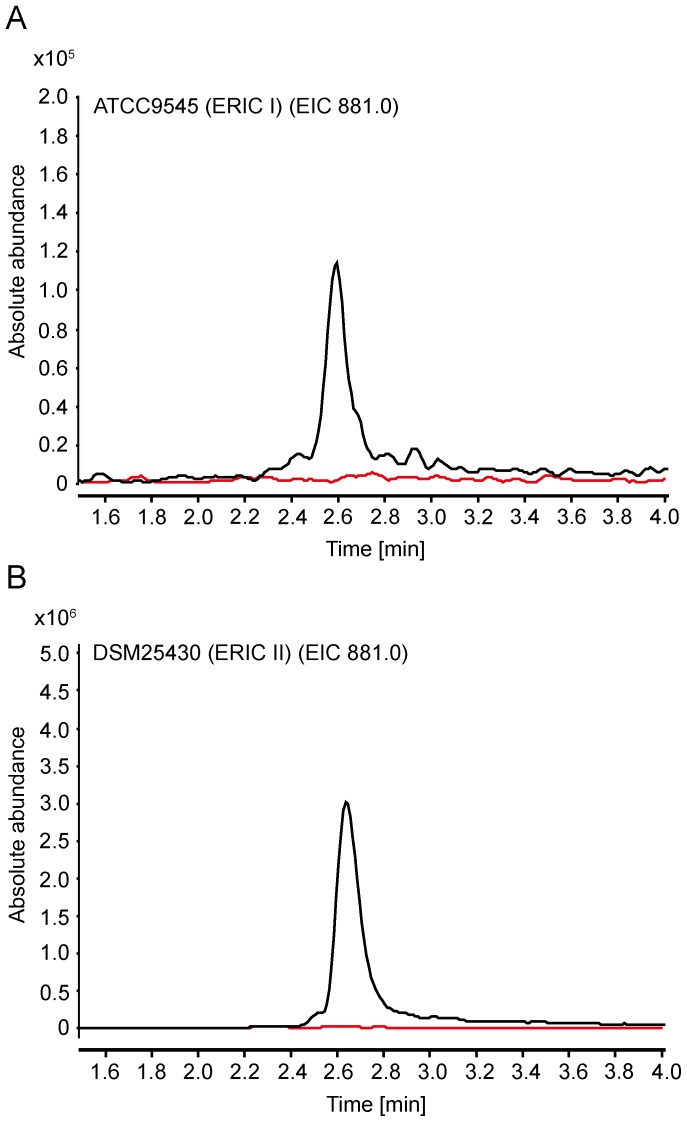
Liquid chromatography (LC) ESI-negative mass spectrometry (MS) analytics of ethyl acetate extracts of *P. larvae* secretomes. (A) Extracted ion chromatogram (*m/z* 881) of ethyl acetate extract *P. larvae* ATCC9545 wildtype (ERIC I; black line) and *P. larvae* ATCC9545 Δ*dhb*F (red line). (B) Extracted ion chromatogram (*m/z* 881) of ethyl acetate extract of *P. larvae* DMS25430 wildtype (ERIC II; black line) and *P. larvae* DMS25430 Δ*dhb*F (red line).

**Figure 5:**
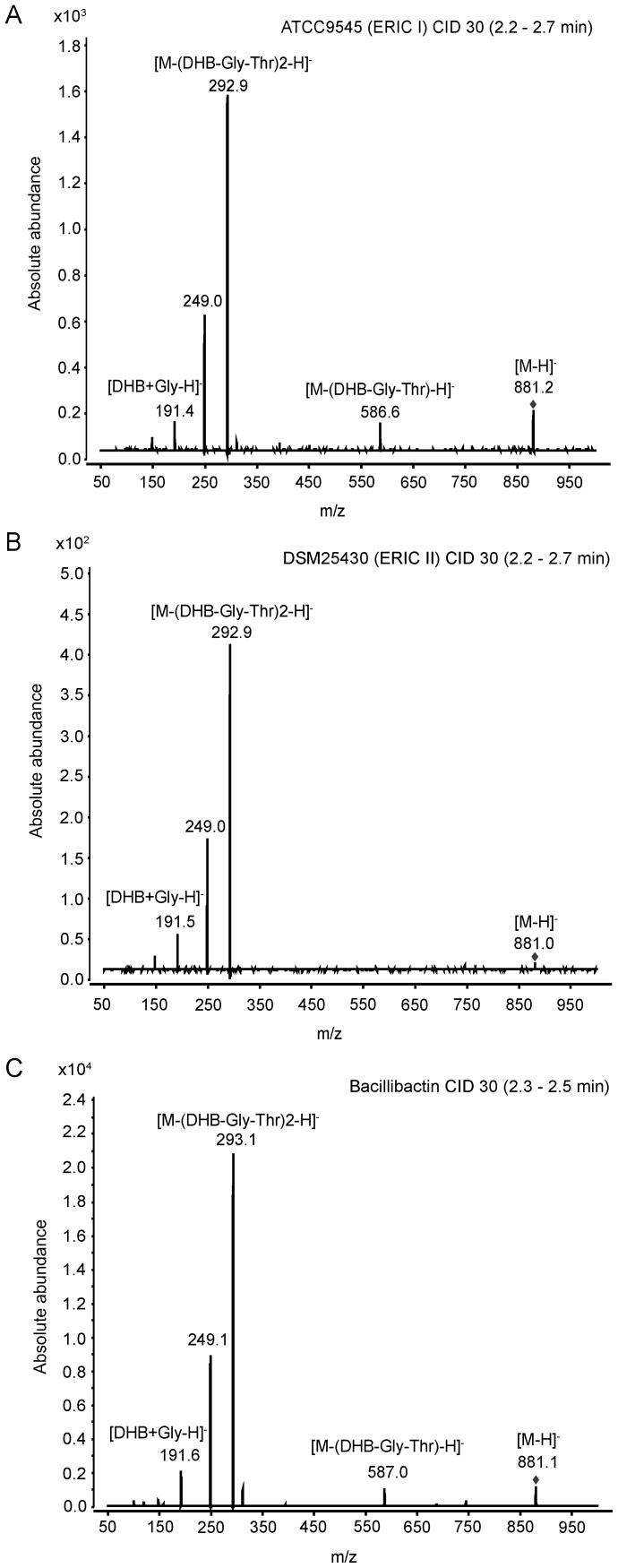
Liquid chromatography (LC) ESI-negative tandem mass spectrometry (MS/MS) analytics of bacillibactin. MS/MS analytics of ethyl acetate extract of *P. larvae* ATCC9545 (A; ERIC I), *P. larvae* DSM25430 (B; ERIC II), and of commercial bacillibactin (C) are shown. The single charged molecular ion of bacillibactin (m/z 881) was chosen for fragmentation with collision-induced dissociation (CID; 30 eV). Fragments are indicated in the spectrum; fragment *m/z* 249.1 is attributed to the decarboxylation of DHB-Gly-Thr.

The *dhb* gene cluster showed considerable similarity to the *dhb* clusters found in several *Bacillus* species. By *in silico* analyses we concluded that the correspondent biosynthesis product will have the same primary structure (DHB-Gly-Thr) as bacillibactin. Therefore, the final confirmation that the siderophore induced in our assays indeed is produced by the *dhb* gene cluster was expected from subsequent experiments aiming for the bioanalytical detection and characterisation of *P. larvae* bacillibactin. To unravel the structure of *P. larvae* bacillibactin, we first extracted the siderophore from the Insect-XPRESS agar by acidification of the agar and subsequent mixing with ethyl acetate. This extract was then analysed by UHPLC-ESI mass spectrometry. *P. larvae* ERIC I and ERIC II wild type strains showed a diagnostic peak in negative ionisation mode (molecular mass [M–H]^−^  =  881.1) corresponding to the negative charged mass of bacillibactin, that were not observed in the *P. larvae Δdhb*F mutant strains (Fig. 4A,B). To verify our assumption, we performed product ion scans of the pseudomolecular ion [M–H]^−^  =  881 Da of the reference compound bacillibactin. The fragmentation patterns of both, the precursor ion from the ethyl acetate extracts from the *P. larvae* wild type strains ATCC9545 (Fig. 5A) and DSM25430 (Fig. 5B) and the reference compound bacillibactin (Fig. 5C), were identical. These results unambigously confirmed that the *dhb* gene cluster is responsible for the expression of the NRPS assembly line producing the catechol-type siderophore, *P. larvae* bacillibactin, with a structure identical to the structure of bacillibactin from other *Bacillus* species as already predicted by *in silico* analyses.

### Role in pathogenesis

**Figure 6:**
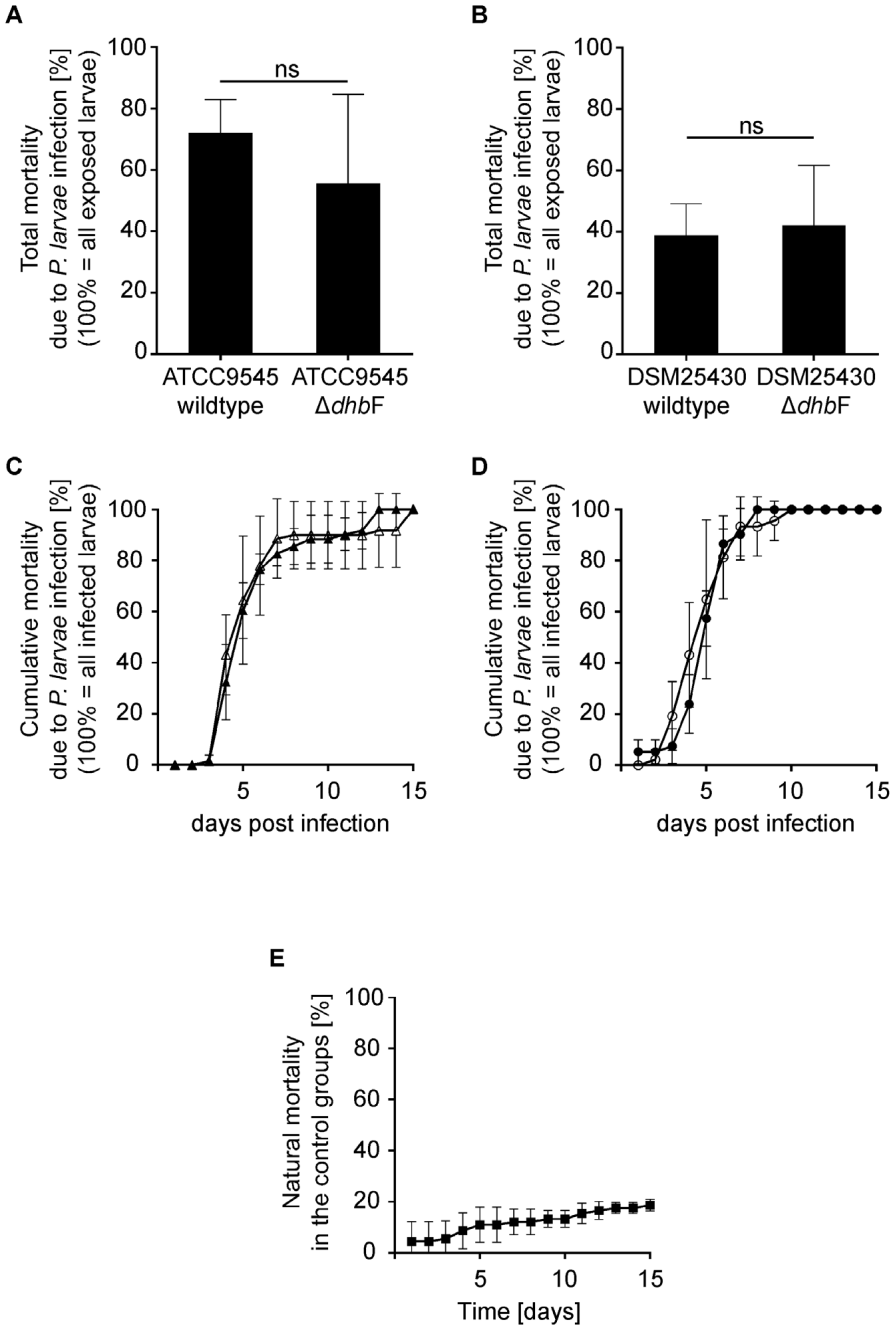
Exposure bioassays for assessing the role of bacillibactin during pathogenesis. Honey bee larvae were infected with wild type *P. larvae* (ATCC9545, DSM25430) or the corresponding mutant strains (ATCC9545 Δ*dhb*F, DSM25430 Δ*dhb*F) and total mortality (A, B) as well as cumulative mortality (C, D) were calculated for each group. Groups with non-infected larvae served as controls (E). All data represent mean values ± SD of three independent infection assays with 30 larvae each. Total mortality due to *P. larvae* infection was not significantly different (Mann–Whitney U test) between ATCC9545 wild type and ATCC9545 Δ*dhb*F (A; p-value = 0.800) and between DSM25430 wild type and DSM25430 Δ*dhb*F (B; p-value = 0.800). Cumulative mortality due to *P. larvae* infection was not significantly different (Kolmogorov–Smirnow test) between ATCC9545 wildtype (C, closed triangles) and ATCC9545 Δ*dhb*F (C, open triangles) (p-value = 0.660) and between DSM25430 wild type (D, closed circles) and DSM25430 Δ*dhb*F (D, open circles) (p-value = 0.999). Natural mortality in the control groups did not exceed 20% (E, closed squares).

Because *P. larvae* bacillibactin played a role for the growth of *P. larvae*, we tested whether this siderophore is a virulence factor during infection of honey bee larvae with *P. larvae*. Exposure bioassays were performed by feeding first instar larvae with spores of *P. larvae* wild type strains DSM25430 and ATCC9545 and of the corresponding mutant strains lacking bacillibactin synthesis (Figs. 3A, 4). Total mortality (Fig. 6A,B) as well as cumulative mortality due to *P. larvae* infection (Fig. 6C,D) were determined and differences between larvae groups infected with wild type and the corresponding mutant strains were analysed statistically. Unexpectedly, no significant changes in total mortality (Mann-Whitney U test) between ATCC9545 wild type and ATCC9545 Δ*dhb*F (p-value = 0.800) and between DSM25430 wild type and DSM25430 Δ*dhb*F (p-value = 0.800) were observed. Also cumulative mortality did not differ significantly (Kolmogorov-Smirnow test) between ATCC9545 wild type and ATCC9545 Δ*dhb*F (p-value = 0.660) and between DSM25430 wild type and DSM25430 Δ*dhb*F (p-value = 0.999). These results suggested that bacillibactin activity is not required for *P. larvae* to be a successful pathogen in laboratory exposure bioassays.

## Discussion

Siderophores are produced by microorganisms mainly in response to a deficiency of iron. Therefore, studies on bacterial siderophore production are best performed with iron-limited or iron-deficient media followed by demonstration of siderophore production using dye-based assays such as the overlaid chrome azurol sulfonate (CAS) agar plate assay for Gram-positive bacteria [49]. However, investigation of *P. larvae* siderophore production was hampered by the fact that *P. larvae* did not grow in established low iron media and no siderophore production was detectable in iron-depleted, established growth media for *P. larvae*. We here present a solution to this problem by demonstrating that an insect cell culture medium (Insect-XPRESS) not only allowed growth of *P. larvae* but that limiting iron availability in this medium triggered the production of a catechol-type siderophore in *P. larvae* structurally identical to bacillibactin. This method not only enabled the identification of *P. larvae* bacillibactin but it will also foster future research on *P. larvae* iron uptake and metabolism.

*In silico* analyses suggested the structure of a trimeric trithreonyl lactone (DHB-Gly-Thr)_3_ for the *P. larvae* siderophore. This structure could be unambiguously confirmed by mass spectrometric analysis. Hence, the *P. larvae* siderophore is structurally identical to bacillibactin, a catechol-type siderophore first described in *B. subtilis* [35], [56], [57] and later shown to be also utilized by members of the *B. cereus sensu lato* group, *B. cereus*, *B. thuringiensis*, and *B. anthracis* [33]. The result, that *P. larvae* produces bacillibactin is contradictory to the recent annotation of the corresponding gene cluster predicting the production of a paenibactin homolog by *P. larvae* [19]. However, paenibactin synthesized by *P. elgii* was shown to be assembled from DHB, alanine, and threonine [36] thereby representing a novel siderophore not identical with bacillibactin.

Trying to elucidate the biological function of *P. larvae* bacillibactin during AFB pathogenesis revealed that neither total larval mortality nor disease progression (cumulative larval mortality) differed as a function of presence or absence of *P. larvae* bacillibactin. Hence, bacillibactin cannot be considered a virulence factor of *P. larvae*. At first sight, this was surprising since siderophore-mediated scavenging of ferric iron from host organisms is thought to contribute significantly to the virulence of pathogenic bacteria [58], [59]. In addition, it was recently demonstrated that disruption of bacillibactin production in *B. cereus* resulted in attenuated bacterial virulence in an insect model system [60]. However, siderophore biosynthesis by itself is not *per se* an indication of pathogenicity or virulence since non-pathogenic bacteria [34] or avirulent strains of pathogenic bacteria [61] also possess siderophore gene clusters and secrete siderophores in response to iron deprivation. In addition, it has been shown that not all siderophores secreted by bacterial pathogens are equally required for full virulence in natural or model hosts. *B. anthracis* for instance contains two predicted siderophore synthesis operons responsible for the production of petrobactin (*asb* gene cluster) and bacillibactin (*dhb* gene cluster). Comparative studies in mice performed with *B. anthracis* mutant strains lacking either bacillibactin or petrobactin synthesis and the parental wild type strain revealed that the petrobactin gene cluster is required for full virulence in mice and is indispensable for survival of *B. anthracis* in the analysed mouse model [62]. In contrast, bacillibactin does not play a significant role in *B. anthracis* mouse virulence [62] although it is involved in iron uptake *in vitro*. An explanation for this phenomenon was recently presented by demonstrating that mammalian siderocalin, described as bacteriostatic agent involved in innate immunity [63], can bind ferric siderophores containing the 2,3-DHBA (bacillibactin and homologs) but not the 3,4-DHBA subunit (petrobactin), thereby impeding uptake of iron by *B. anthracis* through bacillibactin but not through petrobactin [64]. Hence, mammalian innate immune responses can counteract the activity of bacillibactin but not of petrobactin thereby neutralizing the potential virulence function of bacillibactin. Whether a similar mechanism is active in honey bee larvae during *P. larvae* infection is completely unknown because no siderocalin or related innate immunity factor has so far been identified in the honey bee genome. However, it is tempting to speculate that honey bees also evolved a mechanism to obstruct *P. larvae* iron uptake through bacillibactin and, hence, *P. larvae* virulence might already reflect the lack of ferric bacillibactin uptake. Future experiments will have to address this question.

In the vertebrate system, the players in the battle for iron between bacterial pathogens and their hosts are well known. On the one hand, vertebrates evolved means to sequester iron from invading pathogens by the use of iron storage proteins like for instance ferritin or transferrin. Iron bound to these proteins is not easily available to bacterial pathogens and this withholding of iron is part of what is termed nutritional immunity. On the other hand, bacterial pathogens have evolved strategies to survive and thrive in vertebrate tissue although it is basically devoid of free iron. To circumvent iron withholding by vertebrates, pathogenic bacteria evolved high-affinity iron uptake mechanisms like siderophores that overcome host-mediated iron sequestration (for a recent review see [58]). The situation for invertebrates and especially for insect hosts and their bacterial entomopathogens is less well understood. It is known that insects also possess iron storage proteins [65]–[67] and that entomopathogens synthesize NRPS siderophores [33], [68]. However, whether a competition between host iron sequestration and pathogen iron scavenging similar to the vertebrate system exists and whether insects also use withholding of nutrients (nutritional immunity) as first line defense against invading entomopathogens still remains elusive.

Even less than for insects in general is known about iron homeostasis in honey bees and especially in honey bee larvae and nothing about the role of iron during *P. larvae* infection of honey bee larvae. Therefore, the identification of *P. larvae* bacillibactin is only a first step into the direction of elucidating the role of putative larval iron sequestration and *P. larvae* iron scavenging during pathogenesis of *P. larvae* infections.

## Material and Methods

### Bacterial strains and culture conditions

*Paenibacillus larvae* ERIC I strain ATCC9545 and ERIC II strain DSM25430 used in this study were cultivated at 37°C in MYPGP (Mueller-Hinton-yeast-phosphate-glucose-pyruvate) broth and agar plates [14], [69] and Columbia sheep blood agar (CSA) plates [11], [70]. As selective media for gene inactivation strains, MYPGP-agar plates supplemented with 5 µg/µl chloramphenicol were used [16]. To test for siderophore expression several media were used (Table 3): M9 [79], Low iron media (LIM) [51], Luria Bertani (LB) [71], Sucrose salt medium (SSM) [72], Leibowitz-15 (L15) [54], MYPGP, Brain-heart-infusion (BHI) [70], Reasoner's 2A (R2A) [73], BM 3 [54], Schneider's Drosophila Medium (Thermo Fisher, Schwerte, Germany), Sf900 II SFM (Thermo Fisher, Schwerte, Germany), Insect-XPRESS (Biozym, Hessisch Oldendorf, Germany). To establish iron deprivation for *P. larvae*, media were pre-treated with Chelex 100 (Biorad, München, Germany) according to the manufacturer's instructions. Agar plates were prepared by mixing the media with autoclaved 10% agar to a final agar concentration of 2%.

**Table 3:** Primers used for construction of gene knock-outs in *P. larvae* ATCC9545 (ERIC I) and DSM25430 (ERIC II).

Primer name	Primer sequences	Purpose
*dhb*F_F	5′-CTGATATAATCATTGCGGCA-3′	*Knock-out* screening
*dhb*F_R	5′-TGGAAAATATAGCGCTT-3′	wt 524 bp; Δ*dhb*F 1439 bp
IBS_*dhb*F_1418	5′-AAAAAAGCTTATAATTATCCTTAAGCGGCA GTAAAGTGCGCCCAGATAGGGTG-3′	
EBS1d_*dhb*F_1418	5′-CAGATTGTACAAATGTGGTGATAACAGATA AGTCAGTAAATTTAACTTACCTTTCTTTGT-3′	*dhbF* knock-out construct
EBS2_*dhb*F_1418	5′-TGAACGCAAGTTTCTAATTTCGATTCCGCT TCGATAGAGGAAAGTGTCT-3′	

For analysis of growth kinetics, bacteria were re-suspended in Insect XPRESS medium and bacterial suspension was brought to a calculated optical density at 600 nm (OD_600_) of 0.5 and then diluted 1∶100 in the respective medium. Growth in a 96-well microtiter plate (Corning, Wiesbaden, Germany) at 37°C under continuous shaking was monitored for 24 h by measuring OD_600_ every hour in a BioTek^™^ SynergyHT^™^ Multidetection Microplate reader (BioTek, Bad Friedrichshall, Germany).

For the detection of siderophores, bacteria were plated on CSA plates from a glycerine stock. A single colony was spread on Chelex 100 treated Insect-XPRESS agar plates, grown at 37°C for 72 h and analysed by overlay with chrome azurol sulfonate (CAS) agar [49]. Plates were incubated at 37°C in the dark for 2 hours before visual inspection. A colour change from blue to orange indicates presence of siderophore activity.

For assessing *P. larvae* sensitivity to iron levels, 10 µl aliquots of a bacterial suspension (calculated OD_600_ of 0.005) were spotted onto MYPGP agar plates supplemented with increasing concentrations (0 – 800 µM) of the chelator 2,2′-dipyridyl (Sigma, Taufkirchen, Germany). Plates were dried for 15 minutes and then incubated at 37°C for 72 h. Three independent experiments (biological replicates) with three technical replicates each were performed.

Spores of *P. larvae* were generated and concentrations were determined as described previously [14]. *Escherichia coli* DH5α cells (Invitrogen) were treated as described [15], [16], [39] and transformed bacteria containing the plasmid pTT_*dhb*F were cultured on Luria Bertani (LB) broth and agar plates supplemented with 30 µg/µl chloramphenicol as described [15], [16], [39].

### Sequence analysis

The genomes of *P. larvae* ERIC I and ERIC II have been sequenced and annotated [19]. In both genotypes, a gene cluster containing three NRPS domains and putatively coding for a siderophore NRPS has been identified [19]. In *P. larvae* ERIC I (NCBI acc. no. ADFW01000001.1) and ERIC II (NCBI acc. no. NC_023134.1) the corresponding regions span pos. 749,737 to pos. 761,750 and pos. 1,509,130 to pos. 1,524,149, respectively. Sequence similarity searches were performed using BLASTX and BLASTP at the NCBI website [74]. Domain organization, identification of active site residues, and substrate specificity predictions were analyzed using the following two *in silico* tools: NRPSpredictor2 [45], [46] and SBSPKS [44].

### Disruptive intron insertion into the *dhb*F gene of *P. larvae* DSM25430 and ATCC9545 (gene inactivation mutagenesis)

Gene inactivation of *dhb*F in ERIC I (GenBank: ETK27267.1) and ERIC II (GenBank: AHD05401.1) was achieved by targeted insertion of a disruptive group II intron into the *dhb*F open reading frame using a strategy recently established for *P. larvae* [15], [16], [39]. The vector pTT_*wsf*A243 was modified using appropriate primers (Table 3) which were generated by a computer TargetTron algorithm (http://wwww.sigma-genosys.com/targetron) as already described [16]. The obtained vector pTT_*dhb*F was transformed into *E. coli* DH5α for replication and isolation and then used to retarget the LI.LtrB intron at position 878 from the *dhb*F start codon thus interrupting the *dhb*F gene and its expression. Generation of electrocompetent cells and transformation was performed as described previously [75], [76].

### Extraction of siderophore from agar

*P. larvae* strains (DSM25430 wt, DSM25430 Δ*dhb*F, ATCC9545 wt and ATCC9545 Δ*dhb*F) were grown on Chelex 100 pre-treated Insect-XPRESS agar plates at 37°C for 72 h. Subsequently, the plates were cut into very small pieces with the back of a sterile pipette tip. The material obtained from three plates of the same strain was pooled and acidified with 5 ml of 10% H_2_SO_4_. Siderophore extraction was performed by adding 50 ml of ethyl acetate and subsequent vigorous stirring at RT for 2 h. The siderophore containing extract was collected by filtration through Whatman paper (Whatman, Dassel, Germany). For a second extraction, 25 ml of ethyl acetate were added to the agar for 30 min and stirred at RT for 20 min. Both extracts per strain were combined and stored at −20°C until further analysis. As negative control non-inoculated Chelex 100 treated Insect-XPRESS agar plates were used.

### Liquid chromatography-electrospray ionization-mass spectrometry (LC-ESI-MS) analytics

For LC-ESI-MS analytics, the ethyl acetate extracts were dried *in vacuo* and resolved in 15 ml 40% aqueous acetonitrile + 0.1% formic acid and 4 µl were injected onto the UHPLC (ultrahigh performance liquid chromatography) column. LC-ESI-MS analytics were performed using an Agilent6410 Triple Quadrupole LC/MS system in negative ionization mode coupled to an UHPLC 1290 Infinity-Series (Agilent Technologies, Waldbronn, Germany). A VisionHT C18 Column 50 mm×2 mm, 1.5 µm (Grace, Grace GmbH & Co KG, Worms, Germany) was used for separation. Samples were analysed by linear gradient elution using H_2_O + 0.1% formic acid as solvent A and acetonitrile + 0.1% formic acid as solvent B. The gradient was from 5% to 100% solvent B in 5 min with a 1.4 min isocratic elution at 100% for solvent B. Mass spectrometry parameters were optimized with commercial bacillibactin (EMC microcollections GmbH; Tübingen, Germany): fragmentor voltage: 80 V; cell accelerator voltage: 3 V; capillary voltage: 3000 V; nozzle voltage: 500 V. The optimized method was used for product ion scans (collision energy: 30 eV (collision-induced dissociation, CID)) with bacillibactin ([M-H]^−^  =  881 Da) as precursor ion.

### Exposure bioassay

To analyse a possible role of the siderophore during pathogenesis, infection assays were conducted essentially as already described [7], [14], [77]. Briefly, first instar larvae were grafted and reared in 24-well plates at 32°C. For infection, larval diet (3% (wt/vol) fructose, 3% (wt/vol) glucose and 66% (vol/vol) royal jelly) was spiked with a defined spore concentration: For infections with *P. larvae* DSM25430 wildtype or DSM25430 Δ*dhb*F, a final spore concentration of 100 cfu/ml was used; for infections with ATCC9545 wild type or ATCC9545 Δ*dhb*F a final spore concentration of 500cfu/ml spores was used. The infectious larval diet was fed to the larvae for the first 24 h after grafting. Thereafter, normal larval diet was used for feeding until the end of the experiment. Uninfected control larvae were fed pure larval diet without spores throughout the entire experiments. Larvae were monitored daily. Dead larvae were recorded and removed while live larvae were transferred to a new well with fresh larval diet. Upon defecation, larvae were transferred to a well lined out with tissue and monitored until 15 days post infection. Dead larvae were classified as dead from AFB only when vegetative *P. larvae* could be cultivated from the larval remains, as determined by established diagnostic methods [78]. Only AFB dead larvae were incorporated into the calculations and figures. On no occasion was *P. larvae* cultivated from remains of dead control animals. Experiments with a mortality exceeding 20% in the control group were invalidated, as were experiments where the natural mortality (larval death but no growth of *P. larvae*) in the infected groups exceeded 20% (Fig. 6E) [77]. All experiments were performed three times (n = 3) with 30 larvae per experiment. Data was plotted and statistical analysis was performed using GraphPad Prism 6. Normal distribution of the data could not be demonstrated, therefore, non-parametric tests (Mann-Whitney U test for total mortality, Kolmogorov-Smirnow test for cumulative mortality) were used for statistical analyses.
